# Errors in Table 3

**DOI:** 10.1001/jamanetworkopen.2026.27357

**Published:** 2026-07-09

In the Original Investigation titled “Life-Sustaining Treatment Decisions and Parent Caregiving Experiences for Children With Medical Complexity,”^1^ published June 11, 2026, there were errors in Table 3. For the subtheme titled, “Emotional-rational entanglement,” the second quote should have been attributed to a “39-year-old father of 7-year-old child with Goldenhar syndrome,” and the third quote should have been attributed to a “37-year-old mother of 4-year-old twins born prematurely.” In the abbreviations footnote, CMC should have been spelled out as “children with medical complexity.” This article has been corrected.^1^
